# Early prediction of developing spontaneous activity in cultured neuronal networks

**DOI:** 10.1038/s41598-021-99538-9

**Published:** 2021-10-14

**Authors:** David Cabrera-Garcia, Davide Warm, Pablo de la Fuente, M. Teresa Fernández-Sánchez, Antonello Novelli, Joaquín M. Villanueva-Balsera

**Affiliations:** 1 Department of Biochemistry and Molecular Biology and University Institute of Biotechnology of Asturias (IUBA), Campus “El Cristo”, University of Oviedo, 33006 Oviedo, Spain; 2 Department of Psychology and University Institute of Biotechnology of Asturias (IUBA), Campus “El Cristo”, University of Oviedo, Institute for Sanitary Research of the Princedom of Asturias (ISPA), 33006 Oviedo, Spain; 3 Project Engineering Area, University of Oviedo, 33004 Oviedo, Spain; 4 Present Address: Institute of Physiology, University Medical Center of the Johannes Gutenberg University Mainz, Duesbergweg 6, 55128 Mainz, Germany; 5 Present Address: Department of Synapse and Network Development, Netherlands Institute for Neuroscience, 1105 BA Amsterdam, The Netherlands

**Keywords:** Neural patterning, Data mining, Machine learning, Neural circuits

## Abstract

Synchronization and bursting activity are intrinsic electrophysiological properties of in vivo and in vitro neural networks. During early development, cortical cultures exhibit a wide repertoire of synchronous bursting dynamics whose characterization may help to understand the parameters governing the transition from immature to mature networks. Here we used machine learning techniques to characterize and predict the developing spontaneous activity in mouse cortical neurons on microelectrode arrays (MEAs) during the first three weeks in vitro. Network activity at three stages of early development was defined by 18 electrophysiological features of spikes, bursts, synchrony, and connectivity. The variability of neuronal network activity during early development was investigated by applying k-means and self-organizing map (SOM) clustering analysis to features of bursts and synchrony. These electrophysiological features were predicted at the third week in vitro with high accuracy from those at earlier times using three machine learning models: Multivariate Adaptive Regression Splines, Support Vector Machines, and Random Forest. Our results indicate that initial patterns of electrical activity during the first week in vitro may already predetermine the final development of the neuronal network activity. The methodological approach used here may be applied to explore the biological mechanisms underlying the complex dynamics of spontaneous activity in developing neuronal cultures.

## Introduction

Burst activity and synchronization are intrinsic features of neural network development in vivo and in vitro, important for both neuronal communication and information processing^1–3^. Brain neuronal networks in newborn mammals are immature and present similar electrophysiological properties^4^, characterized by a combination of local synchronous patterns^5^. Moreover, specific firing patterns of synchronization and desynchronization are correlated to cognitive processes and pathophysiological conditions^6^. Understanding the relationship between network dynamics and development is crucial to identify the mechanisms underlying the maturation of neural circuits^7^. Cortical neurons in vitro are an effective model to study these mechanisms because neuronal cultures retain some of these network dynamics that can be recorded with the use of microelectrode arrays (MEAs)^8^.

Dissociated neurons on MEAs display sparse spontaneous activity during the first week in vitro that shifts to synchronous firing and bursting activity between the second and third week in vitro^9–12^. This developmental pattern comprehends a complex range of spiking and bursting dynamics that vary between dishes and cultures^10^. Although variability in burst activity and synchronization is partly explained by differences in experimental conditions^13^ and cellular density^10,14^, broad heterogeneity remains even in cortical cultures with standardized conditions^10,15^.

Due to the intrinsic variability of neuronal cultures, standardized mean firing rate (MFR) is the most frequent feature reported for in vitro neuropharmacological evaluation^15,16^. However, the complex behavior of neural networks in vitro is not completely characterized without considering the synchronization and burstiness of spontaneous events. Multiple tools are available for the analysis of these features of neuronal firing activity^17^ and, while gold-standard methods are lacking, some methods provide more robust results. For example, Maximum Interval or logISI are highly consistent burst detectors among different dynamics of spontaneous activity^18^. Likewise, measures of correlation such as the Spike Time Tiling Coefficient (STTC) are efficient in detecting spike train synchrony independently of firing rate^19^. Temporal correlations between the spike trains recorded by each electrode can be also analyzed using graph theory to infer connectivity features of the neural network^20,21^. Thus, the analysis of functional connectivity provides a proxy of the topological formation and organization of neuronal networks on MEAs^21–23^.

Integration of spiking, bursting, synchrony, and connectivity features creates a multidimensional profile of network activity which renders the analysis difficult to tackle with traditional statistical methods. To overcome these limitations, machine learning methods arise as an alternative approach for extracting information from large datasets of neural recordings^24^, as well as for predicting variables^25^. Although the use of machine learning algorithms is widely adopted in other fields of neuroscience^26^, few studies have applied these techniques to the study of neural networks in vitro. Dimensionality reduction and classification methods have been applied to integrate electrophysiological features and to identify developmental stages^27,28^, and unsupervised machine learning methods have been used to cluster burst patterns in mature networks^29^. However, to the best of our knowledge, machine learning has not been previously used to study the broad range of developing network activity in vitro.

While modern machine learning techniques like Deep Neural Networks are powerful methods for neural decoding^30,31^, the use of simpler algorithms provides easier interpretation and fewer overfitting problems^32^. Clustering techniques like Self-Organizing Map (SOM)^33^ have been successfully applied to visualize developmental patterns in organogenesis^34^. Furthermore, machine learning regression algorithms have proved to be effective for the prediction of biological data, including Multivariate Adaptive Regression Splines (MARS)^35^, Support Vector Machines (SVM)^36^, and Random Forests^37^. The application and comparison of these methods to investigate whether the development of neuronal networks in vitro is determined by early patterns of electrical activity remains largely unexplored.

Here, we report an integrated methodological approach for the study of developmental patterns of network activity in dissociated cortical neurons during the first three weeks in vitro. We used MEAs to characterize the spontaneous activity of cortical neurons in culture by measuring 18 electrophysiological features of spikes, bursts, synchrony, and connectivity. Clustering analysis allowed us to examine neuronal networks with similar development of bursting and synchronous activity. Then, we successfully used three machine learning models (MARS, SVM, and Random Forest) to predict the levels of electrophysiological features at the third week in vitro, suggesting that the development of network activity is determined by the early electrical activity of neuronal networks. The methodology presented here may help to identify the biological factors determining the maturation of in vitro neural networks.

## Methods

### Primary cortical cultures

Primary cultures of mouse cortical neurons were obtained from the animal colonies hosted in the Bioterium of the University of Oviedo. The animal procedures used were in accordance with the protocols approved by the Institutional Animal Care and Use Committee of the University of Oviedo. All procedures were also carried out in concordance with the European Communities Council Directive (2010/63/UE), Spanish legislation (RD 53/2013), and ARRIVE guidelines. Primary cultures of cortical neurons were prepared from CD1 mice as previously described^38^, adapting the protocol described for cerebellar neurons^39^. Briefly, the brains from 0 to 2-day postnatal pups were sliced, the cortex was dissected from each slice and dissociated both enzymatically (0.125% papain solution) and mechanically (fire-polished Pasteur pipette) in the presence of DNase I. Cells were resuspended in Neurobasal A growth medium supplemented with 1% B27, 2 mM l-glutamine, and 100 mg/ml gentamicin (NB-B27), and seeded in MEA wells (MultiChannel Systems) previously coated with Poly-l-Lysine (5 μg/ml) to achieve an initial density between 1750 and 3500 cells/mm^2^ (generally described as dense cortical cultures^10^). MEAs were incubated at 37 °C with 5% CO_2_, and 25% of the media volume was substituted with fresh NB-B27 every 3 days after the first week.

### MEA recordings

Extracellular recordings of neuronal spontaneous activity were obtained using standard 60 electrode MEA chips (60MEA200/30iR-Ti-gr), 30 µm electrode diameter spaced by a 200 µm distance, with a MEA1060-Inv-BC amplifier (Multi Channel Systems). Raw analog signals were amplified (bandwidth 1 Hz–3 kHz) and sampled at 25 kHz before being filtered with a 200 Hz high-pass filter (Butterworth second-order). Recordings were performed at 37 °C and MEAs were covered with a MEA-MEM Teflon membrane (ALA Scientific) to maintain CO_2_ conditions outside the incubator. After a 3 min stabilization period, 5 min of spontaneous activity was recorded and spikes were extracted from the filtered electrophysiological signal using a threshold method with the MC_Rack software v.4.6 (Multi Channel Systems, https://www.multichannelsystems.com/software/mc-rack). Spikes that crossed a negative threshold set to 5.5 times the SD of the baseline noise were detected and stored in “mcd” files (MC_Rack). Data files generated by MC_Rack 4.6 were converted into HDF5 file format using MultiChannel DataManager (Multi Channel Systems, https://www.multichannelsystems.com/software/multi-channel-datamanager) and imported to MATLAB 9.8 (The MathWorks Inc, https://www.mathworks.com) or Python 3.7 (https://www.python.org/) through the relative toolboxes (https://github.com/multichannelsystems).

### MEA data analysis

A dataset of 231 recordings between day in vitro (DIV) 6 and 18 was pre-processed to remove inconsistent and low-quality recordings. We included for analysis MEAs with one recording at DIV 6–8 and at least one additional recording between DIV 9–18 with spiking activity (> 3 spikes/min) in 10 or more channels. MEAs containing more than 6 channels with noise (amplitude larger than 500 µV or non-characteristic extracellular biphasic waveforms) and with a decrease of network spike activity bigger than 50% during the second week in vitro were discarded from analysis. With this acceptance criteria, 141 recordings of 47 independent MEA dishes from 12 cortical cultures were included for analysis.

Four main groups of electrophysiological features were analyzed to characterize the spontaneous activity of cortical neurons in vitro: spikes, bursts, synchrony, and connectivity. Mean values of the features were used for the analysis. See Table S1 for a description of the 18 electrophysiological features included in the analysis. We selected three developmental stages to study the changes occurring during the second week in vitro and, in order to balance our dataset, the 141 recordings were grouped in three DIV intervals: 6–8 (n = 50), 9–12 (n = 30), and 13–18 (n = 61). Spike analysis was performed in Neuroexplorer 5 (Nex technologies, https://plexon.com/products/neuroexplorer/) for the number of channels with spikes (Ch. spikes), the mean firing rate (MFR), network spikes/s (Network spikes), and interspike interval (ISI). Bursts were analyzed using the maximum interval method in Neuroexplorer 5 (Nex technologies) to obtain the number of channels with bursts (Ch. bursts), mean bursting rate (MBR), Burst duration, network bursts/min (Network bursts), interburst interval (IBI), percentage of spikes in bursts (Burst % spikes), interspike interval in bursts (Burst ISI), peak frequency in bursts (Burst PeakFreq), and burst surprise detection (Burst surprise). We defined a burst by the following parameters: the maximum initial ISI to start the burst (0.1 s), the maximum ISI to define the burst end (0.2 s), the minimum interval between bursts (0.2 s), the minimum burst duration (0.003 s), and the minimum number of spikes in the burst (3).

Synchronization between pairs of electrodes was analyzed with the spike time tiling coefficient (STTC) method^19^ using a Python repository^40^. Briefly, the STTC is calculated as $STTC=\frac{1}{2} \left(\frac{{P}_{A}-{T}_{B} }{1 - {P}_{A}{ T}_{B} } + \frac{{P}_{B}-{T}_{A} }{1 - {P}_{B}{ T}_{A}}\right)$, where P_A_ is the proportion of spikes in channel A that occur within ∆t of a spike from channel B, and T_A_ is the proportion of the total recording time in channel A that falls within ∆t of a spike from channel A. P_B_ and T_B_ are calculated similarly. We considered a predefined time window of 100 ms to quantify the correlation between pairs of spikes trains, and the mean value of STTC was calculated from the square matrix. Then, to identify clusters of synchronized neurons, we used the density-based spatial clustering of applications with noise (DBSCAN) algorithm^41^ on STTC matrices (DBSCAN STTC) implemented in the Python library Sklearn^42^, using eps = 0.2 (eps is the maximum distance between two points to be considered as neighbors, i.e., highly synchronized pairs of electrodes with STTC > 0.8), and 3 as the minimum number of samples to be considered as a core point.

Connectivity parameters were computed and connectivity matrices were generated using the Brain Connectivity Toolbox^43^ in MATLAB 9.8 (The MathWorks Inc). First, spike time series were binned in 100 ms time windows for each electrode, and the spike count correlations were computed as the Pearson correlation coefficient for all pairs of electrodes. Only significant correlations (*P* < 0.05) were retained in the correlation matrices. Then, an absolute weight threshold of 0.35^22^ was applied to discard spurious connections and to obtain undirected connectivity matrices, in which nodes (electrodes) are linked by binary edges (connections), and the following network parameters were extracted: average node degree (Node degree), clustering coefficient (Clustering coeff), and global efficiency (Efficiency) (see Table S1 for definitions).

### Machine learning

To examine the distribution of neuronal networks by developmental stage in a low-dimensional projection, we performed principal component analysis (PCA) with the above 18 electrophysiological features using the R package FactoMineR^44^. Values were scaled to unit variance and the 18-dimensional feature vector was projected onto the first two principal components (PCs) to display data points corresponding to DIV intervals (Fig. 2) and clustering analysis (Fig. S3).

To identify developmental clusters of individual neuronal networks (MEA dishes), we applied the SOM technique^33^ using the R package Kohonen as previously described^45^. Briefly, SOM is a self-organizing neural network with an input and output layer, such that the output cells are activated as a function of the input data. For the 47 independent MEA dishes used in the study, the mean values of the electrophysiological features Ch. bursts and STTC at each DIV interval were taken as inputs to the SOM network. The network competes to activate a unique output cell by every input, which results in a learning model that categorizes similar cases by activating the same output cell. The output layer is visualized as a two-dimensional map of hexagonal cells, where each cell is associated with the centroid of the input cases that activate the cell. SOM clustering algorithm was applied to centered and scaled datasets using random initialization with 50 iterations and the total number of units in the competition layer was set to 25 (5 × 5). The size of the network topology was estimated using a heuristic function and 5 × 5 was assigned as the first level of abstraction. To cluster the neuronal networks (MEAs) with similar behaviors, the k-means clustering technique was applied to the results obtained with the SOM technique. The number of clusters with the best fit (k = 3) was adjusted with the Davies-Bouldin validity index^46^. Mean, median, standard error of the mean (SEM), and interquartile range (IQR) were calculated for the 18 electrophysiological features in each cluster. Additionally, PCA was performed as described above on the data points at DIV 6–8 and DIV 13–18, and clusters defined by k-means and SOM analysis were displayed on the principal components projections.

Prediction of the continuous variables STTC, Ch. bursts, and MFR, was assessed using three robust and optimized machine learning techniques for regression: MARS^47^, SVM^48^, and Random Forests^49^ through the R packages Earth, e1071, and randomForest, respectively. We selected these methods, with distinct learning approaches, to determine whether the prediction of the electrophysiological features was affected by the machine learning model. MARS is a flexible and fast nonparametric regression technique used to capture the non-linear relationships between the variables. MARS (R package Earth) was performed with 5 degrees of interaction and 35 model terms. Random Forest combines multiple decision trees and averages the output, and it is considered to be a good model to control overfitting and select robust features for the model applied^49^. Random Forest was performed with 100 trees, and 8 variables were randomly sampled as candidates at each split. SVM is a robust classifier that handles noisy data and identifies non-linear relationships efficiently^36^. The SVM constructs a hyperplane and creates boundaries to classify values in this high-dimensional space. SVM was implemented using a radial basis function kernel sigma = 0.125 and C = 6. In all cases, 75% of the dataset was used as a training set and 25% as a test set. Regression models were evaluated by the R^2^ coefficient (accuracy) and the root squared mean error (RMSE). For visualization purposes, Regression Error Characteristic (REC) curves^50^ were estimated as the relation between the standardized error on the x-axis and the accuracy of the prediction method on the y-axis. Then, we used the SVM model to quantify the relative importance of the features for the predictive machine learning model. We balanced each group of electrophysiological features using two features per group: spikes (Ch. spikes, MFR), bursts (Ch. bursts, MBR), synchrony (STTC, STTC DBSCAN), and connectivity (Clustering coeff, Efficiency). We designed a “leave-one-in” strategy, in which only one group of variables was left and the results achieved by the model calculated, and, conversely, a “leave-one-out” which consisted of removing only one group of variables. To ensure the robustness of the training and test datasets, we applied an oversampling method to the dataset based on the Synthetic Minority Oversampling Technique (SMOTE)^51^ using the R package smotefamily. Briefly, SMOTE finds the k nearest neighbors (k = 20), selects a random neighbor, and creates a new synthetic instance between the feature space (in this step the minority class is not separated, and all samples were processed). This technique was applied with a ratio of 2 as desired synthetic instances over the original instances and then we evaluated the performance of the SVM model using fourfold cross-validation with 10 iteration cycles to minimize overfitting. Mean R squared (R^2^) of the out-fold (not selected for training) was used as the indicator of test fit goodness and error bars were computed using the SEM. All R packages are available at the CRAN repository (https://cran.r-project.org).

### Statistical analysis

Statistical analyses were performed using Prism 8.0 (GraphPad Software, Inc, https://www.graphpad.com) and R 4.03 (The R Project for Statistical Computing, https://www.r-project.org/). Summary data and graphs were presented as mean ± SEM, median and Interquartile range (IQR), or Tukey box plots. Significance levels were defined at *P* < 0.05. Additional detailed statistical information is provided in the figure legends.

## Results

### Synchronized network activity emerges during early development of cortical neurons in vitro

**Figure 1:**
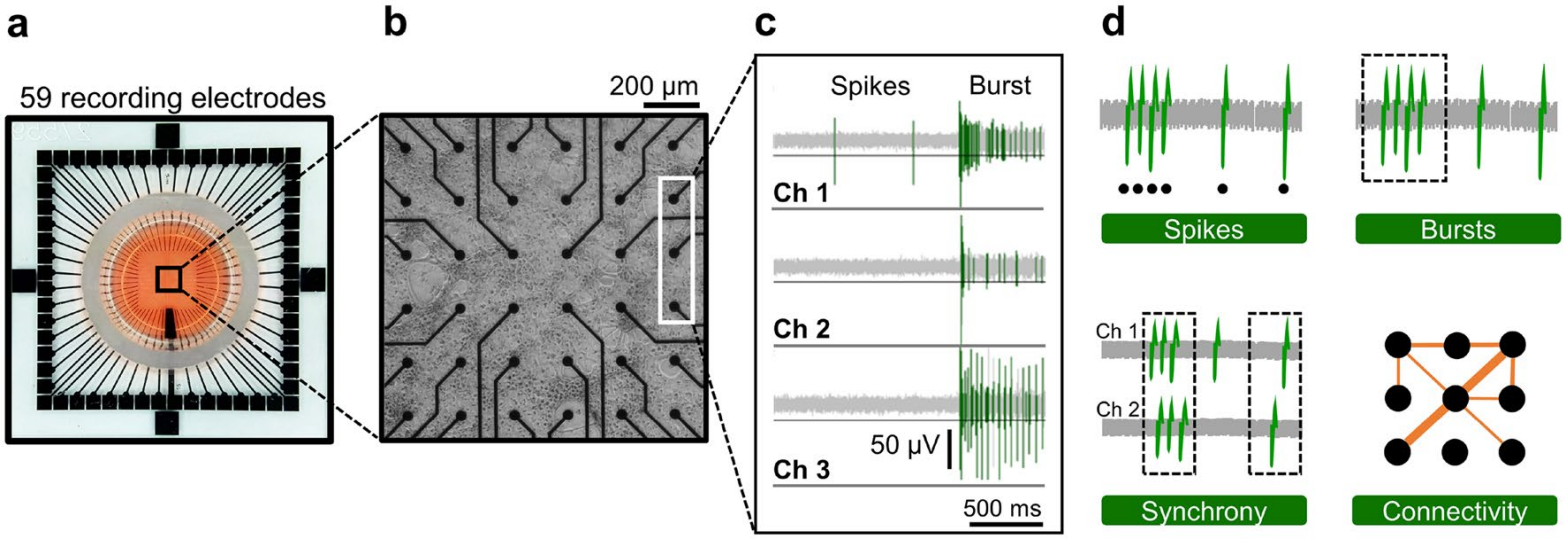
Electrophysiological characterization of cortical neuron cultures on MEAs. (**a**) Photograph of a 60 electrode MEA device used in the study. (**b**) Phase-contrast image of cortical neurons in culture at DIV 14. The distance between electrodes is 200 µm. Each black dot corresponds to one of the recording electrodes. (**c**) Sample traces of spontaneous electrical activity recorded by three channels. Electrical events, spikes and bursts, that crossed a threshold (horizontal black line) were recorded. (**d**) Spontaneous activity of cortical neurons was analyzed according to electrophysiological features of spikes, bursts, synchrony, and connectivity. The figure was created using MC_Rack 4.6 (https://www.multichannelsystems.com/software/mc-rack) (**c**) and Microsoft PowerPoint 365 (https://www.microsoft.com/powerpoint) (**d**).

The spontaneous activity of cortical neurons in culture was recorded with MEAs (Fig. 1a, b) during the first three weeks in vitro. The extracellular electrical signals (spikes) crossing a negative voltage threshold were recorded (Fig. 1c) and 18 features of spikes, bursts, synchrony, and connectivity (see Table S1) were analyzed post hoc to characterize the network activity (Fig. 1d).

**Figure 2:**
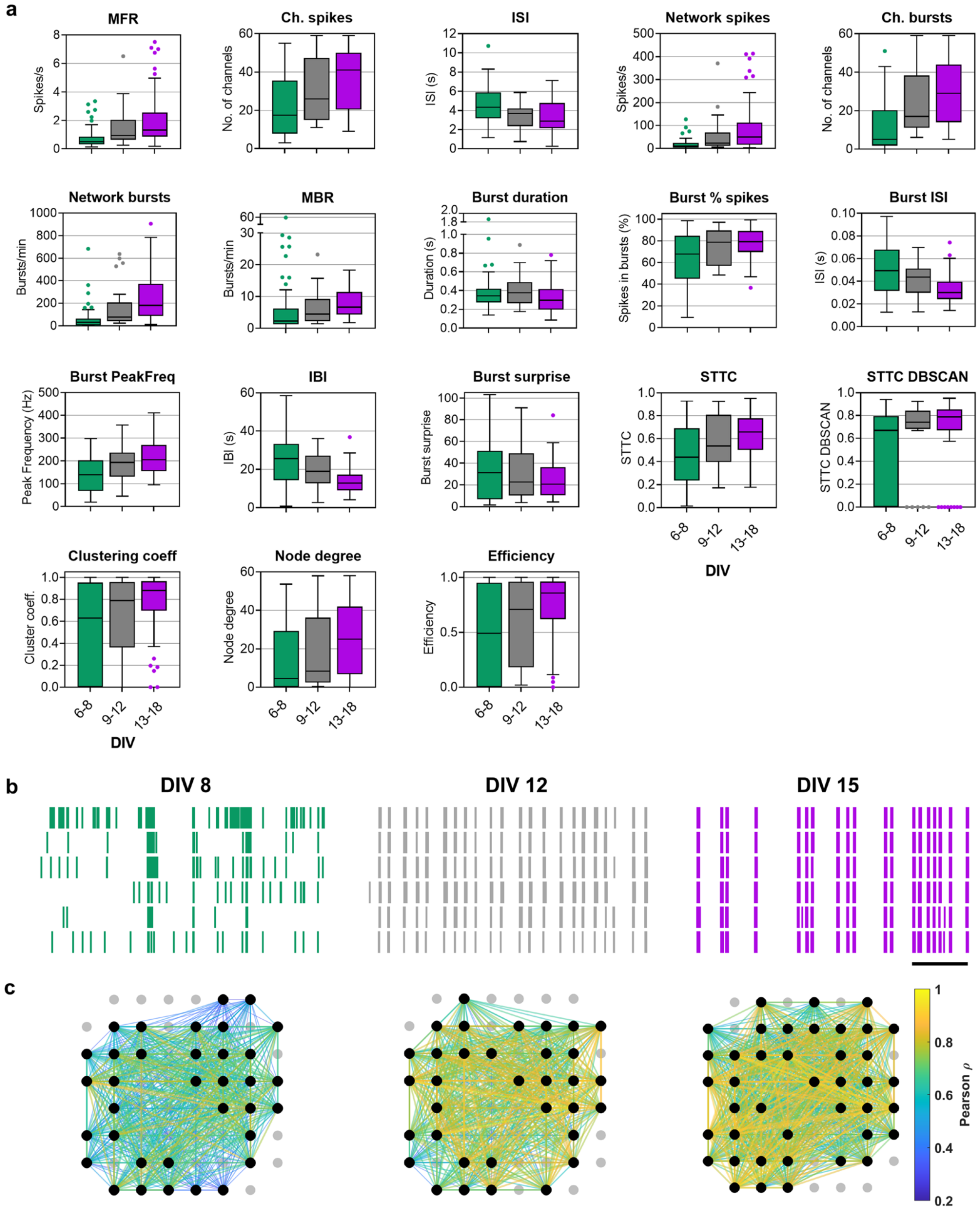
Development of spontaneous activity of cortical neurons in culture during the first three weeks in vitro. (**a**) Analysis of 18 electrophysiological features of neuronal network activity at DIV intervals: 6–8 (n = 50), 9–12 (n = 30), and 13–18 (n = 61). The box plots show the median and the interquartile range with Tukey whiskers. Description of features is provided in Table S1 and *p*-values for (**a**) are reported in Table S2. **(b**) Raster plots show the spontaneous activity recorded by 6 electrodes from a representative neuronal network at DIV 8, 12, and 15 with an archetypical increase of spontaneous activity and synchrony with days in culture. Vertical lines correspond to spikes. Horizontal scale bar, 20 s. (**c**) Maps of functional connectivity of the same neuronal network and period as in (**b**). The color of the connections (edges) between electrodes (nodes) represents Pearson’s correlation and shows a general increase in connectivity during early development. The figure was created using Graphpad 8.0 (https://www.graphpad.com) (**a**), Neuroexplorer 5 (https://plexon.com/products/neuroexplorer/) (**b**), and Matlab 9.8 (https://www.mathworks.com/products/matlab.html) (**c**).

The values of the 18 electrophysiological features analyzed from MEA recordings were grouped in three DIV intervals (6–8, 9–12, and 13–18) which captured the changes occurring from the first to the third week in vitro (Fig. 2a, Table S2). During the first three weeks in vitro, spiking activity increased from a median MFR of 0.5 [IQR 0.3–0.9] spikes/s at DIV 6–8 to 1.3 [IQR 0.8–2.6] spikes/s at DIV 13–18 while the ISI decreased accordingly (Fig. 2a). The increase in spiking activity was also reflected in a higher number of channels registering spikes at DIV 13–18 (Fig. 2a). Bursts, i.e., intermittent groups of high-frequency spikes, are a key feature of network activity in vitro and were analyzed with the maximum interval method which has a high performance in detecting different types of bursts^18^. A maximum interval of 100 ms was defined to start a burst for consistency with the rest of the measures and within the range of previous studies^18,52,53^. Analysis of bursts showed that the median network burst activity had a sixfold median increase (30 to 180 network bursts/min) from the first to the third week in vitro (Fig. 2a). The percentage of spikes in bursts also increased across development and, consequently, a decrease in ISI in bursts, burst peak frequency, and burst surprise was observed during the same period (Fig. 2a). Whereas the variability and median length of IBI decreased approximately by half during the second week in vitro (25.6 [14.3–33.3] s at DIV 6–8 vs 12.8 [9–17.2] s at DIV 13–18), only a slight decrease in the median burst duration was observed (0.34 s at DIV 6–8 vs 0.30 s at DIV 13–18) (Fig. 2a).

STTC index^19^ was used to analyze the level of synchronization of spontaneous activity. The average synchrony obtained with 100 ms bin showed a strong correlation with the percentage of spikes in bursts and within the range of average burst duration (Fig. S1a,b). We observed that levels of STTC and synchronized firing patterns increased from DIV 6–8 to 13–18 (Fig. 2a, b), as well as the number of highly synchronized groups of neurons revealed by applying DBSCAN clustering algorithm to STTC (STTC DBSCAN) results (Fig. 2a). Functional connectivity of neuronal networks on MEAs was analyzed using graph theory^21^, and cross-correlation matrices were calculated over 100 ms bins with an absolute threshold of 0.35 to capture a relevant range of network integration (Fig. S1c), in concordance with previous studies^22^. During the first week in vitro, there was high variability in the levels of Clustering coeff and Efficiency, indicators of network’s levels of segregation and integration^43^, respectively (Fig. 2a). The levels of Node degree increased during the second week in vitro (Fig. 2a, c), and high median levels of Clustering coeff (0.88) and Efficiency (0.86) were representative of neuronal networks at DIV 13–18.

### Dimensionality reduction analysis for integrating electrophysiological features of neuronal network activity

**Figure 3:**
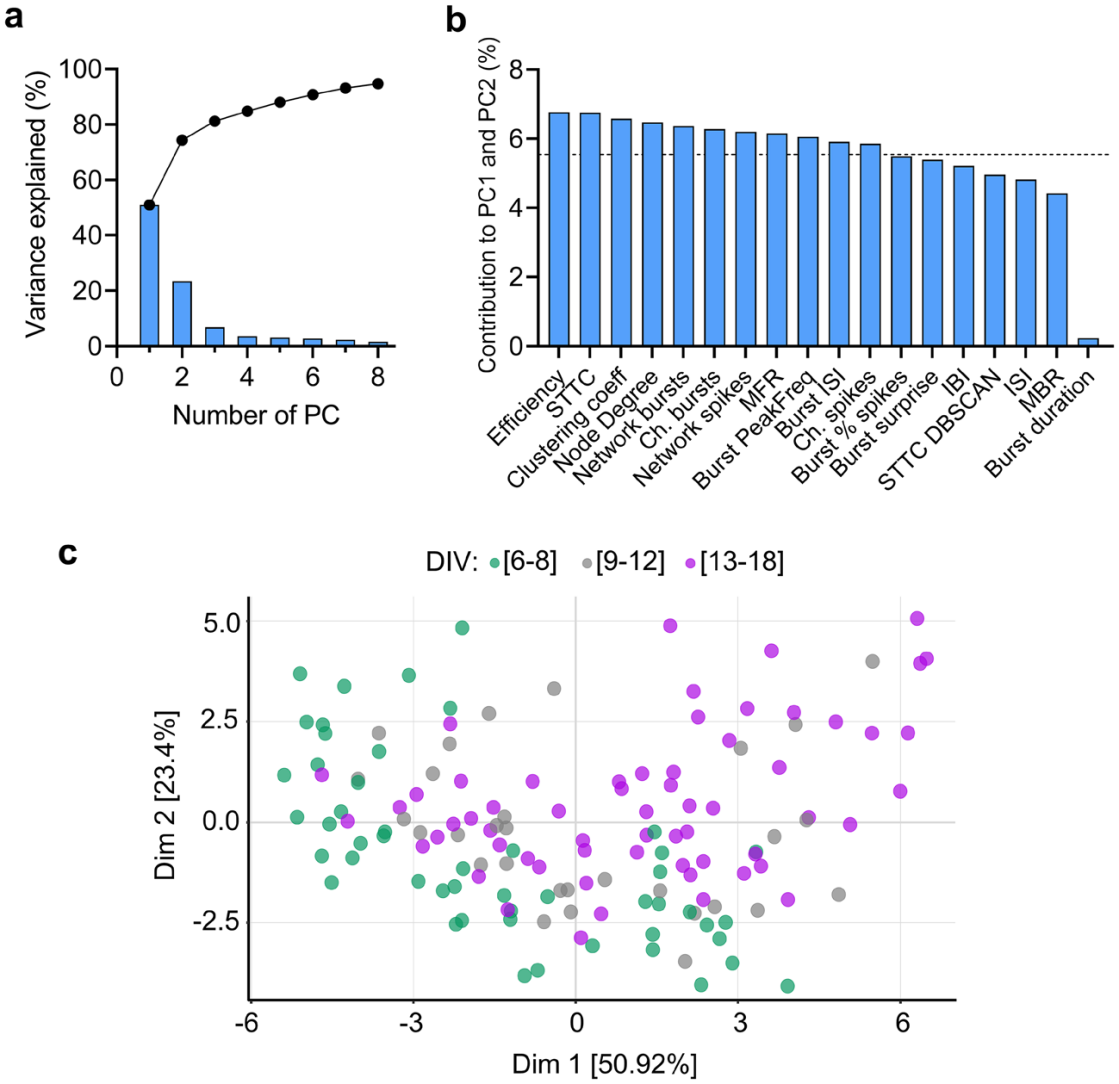
Principal Component Analysis (PCA) of electrophysiological features of neuronal network activity in vitro. (**a**) The graph shows the percentage of variance explained by each principal component (PC) (bars) and the cumulative percentage of variance (dots) by the number of PCs. (**b**) Bar plot displays the contribution (%) of each of the 18 electrophysiological features to the PC1 and PC2. The dotted line represents the significant contribution value (5.55%) for the first two PCs. (**c**) Scatter plot of MEA recordings grouped by DIV intervals in the 2-dimensional PCA based on the 18 electrophysiological features. PC 1 (x-axis) and PC2 (y-axis) captured 74.3% of the total variance and each dot represents a time point recording within each DIV interval: 6–8 (green), 9–12 (gray), and 13–18 (purple) DIV. A gradient from young (DIV 6–8) to mature neuronal networks (DIV 13–18) is captured by the PCA projection: 38.3% of DIV 13–18 recordings are in the positive plane of PC1 and PC2 dimensions while no DIV 6–8 recordings are in the same PC area. The figure was created using Graphpad 8.0 (https://www.graphpad.com) (**a**,**b**), and R 4.03 (https://www.r-project.org/) (**c**).

To explore how the 18 electrophysiological features defined the network activity of cortical neurons in each DIV interval, we conducted a PCA as a method to capture the greatest variance in a low-dimensional projection. The lower-dimensional space defined by the first two PC dimensions accounted for 74.32% of the variance (Fig. 3a). The electrophysiological features that better characterized the variance in the first two PCs were related to network features such as Network bursts, Efficiency, and STTC (Fig. 3b). Despite the lack of clear segmentation between DIV intervals due to the variability within each developmental stage, the first dimension of the PCA captured a left to right gradient from DIV 6–8 to DIV 13–18 (Fig. 3c). A similar distribution of MEA recordings by DIV intervals persisted when the PCA was applied to each cortical culture (Fig. S2a). Moreover, the variability between and within cultures did not seem to depend upon the range of cell densities used in our study (Fig. S2a–c).

### Clustering analysis to explore developmental patterns in neuronal networks in vitro

**Figure 4:**
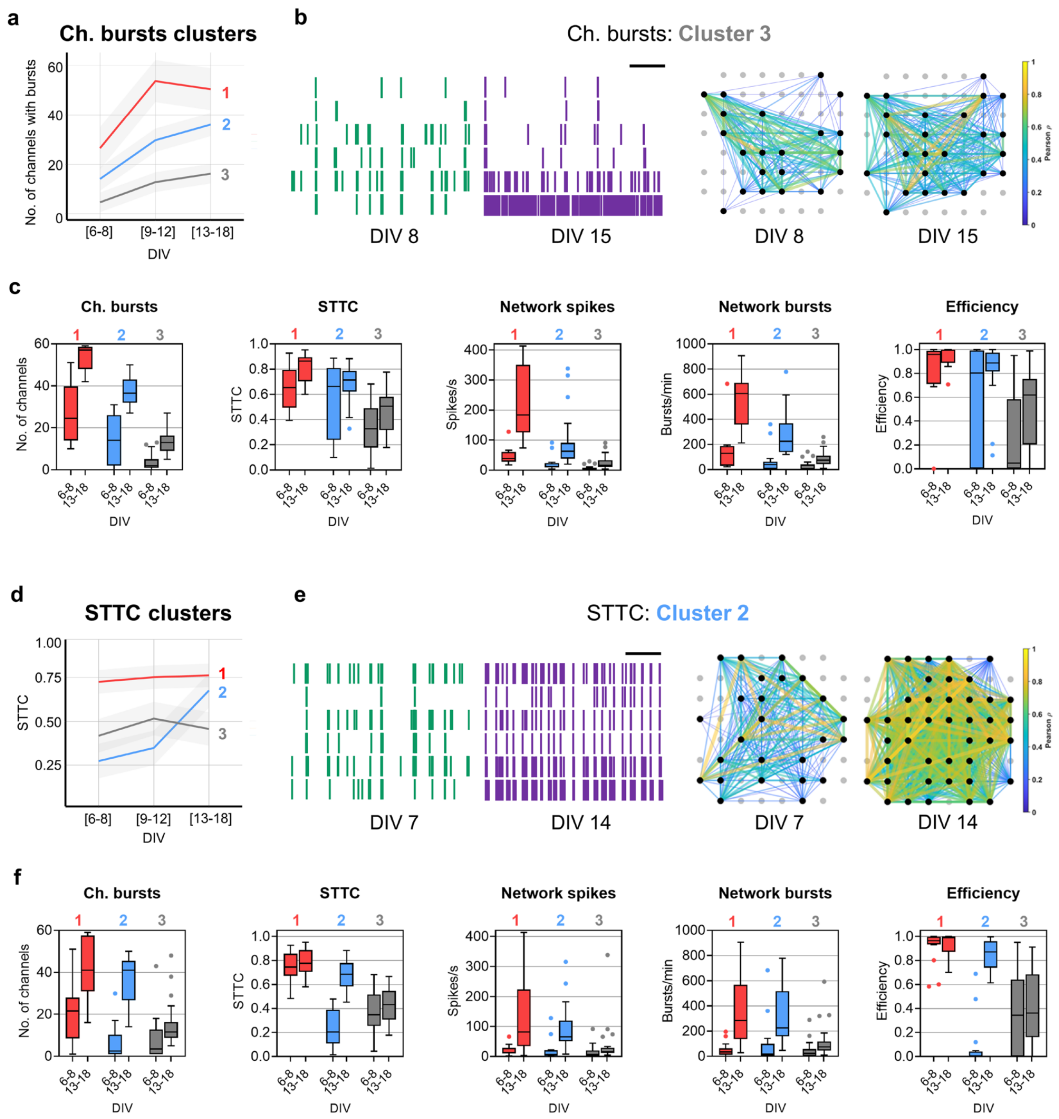
Patterns of developing spontaneous activity in neuronal networks using clustering analysis. (**a**,**d**) Graphs display three developmental patterns for the features of Ch. bursts (**a**) and STTC identified by k-means clustering of the SOM. The lines represent the means of the observations, labelled by k-means, and the shaded areas represent the 95% confidence intervals. (**b**,**e**) Examples of raster plots (left panels) and connectivity maps (right panels) of neuronal networks included in either cluster 3 of Ch. bursts (**b**) or cluster 2 of STTC (**e**). Horizontal scale bar in raster plots, 20 s. Color of the edges between nodes in connectivity maps represents Pearson’s correlation. (**c**,**f**) Changes in Ch. bursts, STTC, Network spikes, Network bursts, and Efficiency between DIV 6–8 and 13–18 in each cluster of Ch. bursts (**c**) and STTC (**f**). The box plots show the median and the interquartile range with Tukey whiskers. Clusters are indicated on top of the graphs. The figure was created using Graphpad 8.0 (https://www.graphpad.com) (**c**,**f**), Neuroexplorer 5 (https://plexon.com/products/neuroexplorer/), Matlab 9.8 (https://www.mathworks.com/products/matlab.html) (**b**,**e**), and R 4.03 (https://www.r-project.org/) (**a**, **d**).

We used clustering techniques to explore whether it was possible to characterize individual neuronal networks with similar developing network activity within the range of activity levels found in our dataset. To do this, we applied k-means clustering at the centroids of the SOM^33^ on two representative network features of synchrony and bursting activity: STTC and Ch. bursts, respectively. For the 47 MEA dishes used in this study, the matrix of the SOM algorithm showed 25 nodes in a hexagonal grid, in which each node represented neuronal networks with similar network activity development (Fig. S3a, b). Then, the k-means analysis of the SOM revealed 3 clusters of neuronal networks with different patterns of development for the parameters of Ch. bursts (Fig. 4a) and STTC (Fig. 4d). We then quantified the 18 electrophysiological features at DIV 6–8 and DIV 13–18 in the neuronal networks included in each cluster (see Table S3).

Clusters 1 to 3 of Ch. bursts showed neuronal networks with developmental patterns from high to low number of channels with burst activity (Fig. 4a) that highly correlated with the changes shown in representative network features of spikes, bursts, synchrony, and connectivity from DIV 6–8 to DIV 13–18 (Fig. 4c). MEAs included in cluster 1 of Ch. bursts were characterized by higher levels of STTC and connectivity at DIV 6–8 as well as the highest increase of network spikes and bursts from DIV 6–8 to DIV 13–18 (Fig. 4c). Conversely, networks with a low number of channels with bursts at DIV 6–8 (cluster 3) evolved into poorly synchronized and integrated networks (Fig. 4b, c).

Clustering based on the synchrony feature STTC included neuronal networks that kept either high (cluster 1) or low (cluster 3) levels of STTC during the three developmental stages, and a group of neuronal networks (cluster 2) with a high increase of synchrony from DIV 6–8 to DIV 13–18 (Fig. 4d). Whereas the changes from DIV 6–8 to DIV 13–18 in the electrophysiological features of neuronal networks included in clusters 1 or 3 of STTC were relatively similar to the equivalent clusters of Ch. bursts (Fig. 4c), neuronal networks included in cluster 2 of STTC were characterized by drastic increases in the levels of STTC and Efficiency from DIV 6–8 to DIV 13–18 (Fig. 4e, f).

The maximum congruence between clustering methods occurred in neuronal networks included in cluster 3 of STTC and Ch. burst; clusters 1 and 2 of Ch. bursts had a good correlation with cluster 1 of STTC (high levels of STTC at DIV 13–18), but 50% of neuronal networks included in cluster 2 of STTC were also included in cluster 3 of Ch. bursts (Fig. S3c). Additionally, PCA analysis performed on the 18 electrophysiological features of MEA recordings at DIV 6–8 and DIV 13–18 showed that the correlation between electrophysiological features was different at each DIV interval (Fig. S3d), and separation of neuronal networks included in clusters of Ch. bursts (Fig. S3e) and STTC was less clear in the PCA projection (Fig. S3f).

Altogether, these results suggest that clustering methods were effective in characterizing neuronal networks with similar changes in spontaneous activity during early development.

### Prediction of levels of synchrony and bursting activity in cultured cortical neurons using machine learning

**Figure 5:**
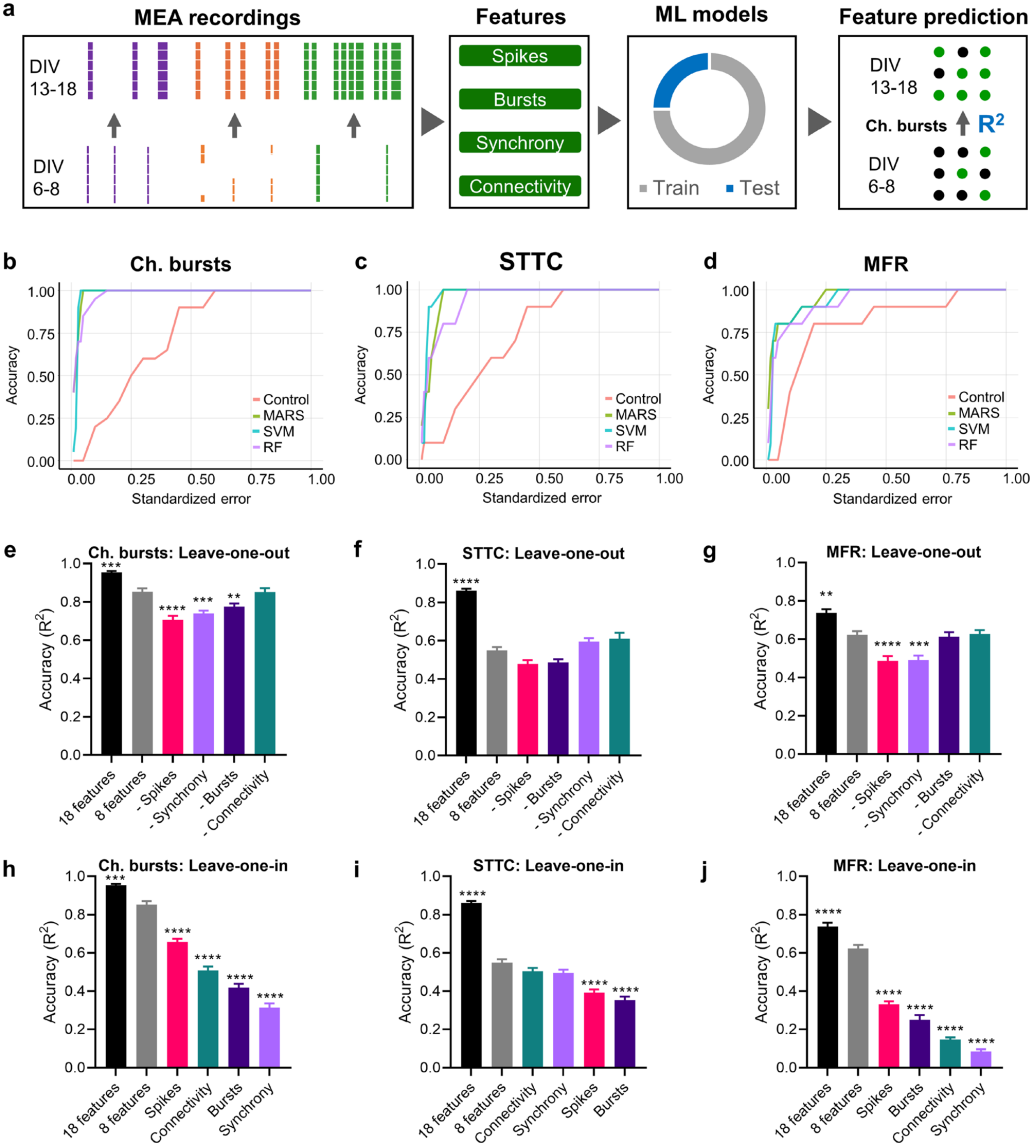
Machine learning for the prediction of features of bursts, synchrony, and spikes. (**a**) Schematics of the machine learning workflow. The 18 electrophysiological features were analyzed from MEA recordings at DIV 6–8 and DIV 13–18 and these features were used to train (gray segment) machine learning (ML) models. Then, accuracy prediction (R^2^) at DIV 13–18 was evaluated in the test dataset (blue segment) for electrophysiological features (e.g., Ch. bursts). Figure created with Microsoft PowerPoint 365 (https://www.microsoft.com/powerpoint). (**b**–**d**). REC curves for control and MARS, SVM, and Random Forest (RF) machine learning models for the prediction of STTC (**b**), Ch. bursts (**c**), and MFR (**d**). REC curves represent the cumulative error obtained by the machine learning models. (**e-j**) SVM prediction of Ch. bursts (**e**–**h**), STTC (**f**–**i**), and MFR (**g**–**j**) using leave-one-out (**e**–**g**) and leave-one-in (**h**–**j**) strategies. We performed fourfold cross-validation (10 iterations) to calculate the mean accuracy of the SVM prediction using 8 electrophysiological features (2 features per group): MFR and Ch. spikes (spikes), MBR and Ch. bursts (bursts), STTC and DBSCAN STTC (synchrony), and Efficiency and Clustering coeff (connectivity). Feature importance was evaluated by removing (leave-one-out) or leaving (leave-one-in) one group of features. Baseline accuracy of cross-validation was calculated using the 18 features. Error bars represent the mean ± SEM. *P < 0.05, **P < 0.01, ***P < 0.001, ****P < 0.0001 vs accuracy obtained with 8 features (one-way ANOVA, followed by Dunnett’s post hoc multiple comparison test). The figure was created using Microsoft PowerPoint 365 (https://www.microsoft.com/powerpoint) (**a**), R 4.03 (https://www.r-project.org/) (**b**), and Graphpad 8.0 (https://www.graphpad.com) (**e**–**j**).

To further investigate the importance of early network activity suggested by the clustering results, we used machine learning techniques to predict the levels of Ch. bursts and STTC at the third week in vitro. These two parameters were compared with the prediction of MFR, one of the most commonly used parameters in MEA studies^15^. The dataset with the 18 electrophysiological features from MEA recordings at DIV 6–8 and DIV 13–18 was divided into training and testing datasets. After training each machine learning model, the performance of the models was evaluated based on the accuracy prediction (R^2^) of the features at DIV 13–18 in the test dataset (Fig. 5a).

We used three machine learning methods with different learning algorithms: MARS, SVM, and Random Forest. The three models yielded predictive accuracies higher than 0.9 during testing for the features of Ch. bursts and STTC, and higher than 0.8 for MFR (Table S4, and Fig. S4 for the SVM model). Additionally, comparison of model performance with REC curves showed that MARS and SVM models performed slightly better than Random Forest for Ch. bursts and STTC, and similarly for MFR (Fig. 5b–d). In all machine learning models, Ch. spikes and ISI appeared to be the most important electrophysiological features for the prediction of Ch. bursts and MFR, respectively, while the rest of the features had relative importance depending on the model (Table S5). Also, features of functional connectivity had the highest relative importance for the prediction of STTC values in all machine learning models (Table S5). These results show the consistency for the prediction of electrophysiological properties of cortical neurons in culture across machine learning models.

To further identify which electrophysiological features were most informative for the prediction of Ch. bursts, STTC, and MFR, we tested the predictive accuracy of the features in the SVM model using cross-validation leave-one-out and leave-one-in strategies. To balance the number of features, we tested these strategies with 2 parameters in each of the four groups of electrophysiological features: spikes, bursts, synchrony, and connectivity (Fig. 5e–j). In the leave-one-out strategy, one group of features was removed each time, and in the leave-one-in, only one group of variables was left each time. Predictive accuracy with SVM for Ch. bursts was higher than 0.8 when the number of electrophysiological features was reduced from 18 to 8 (Fig. 5e), while accuracy dropped to 0.6 or lower for MFR and STTC, respectively (Fig. 5f, g). Evaluation of feature importance showed that when leaving out only one group of two features, prediction performance for the 3 variables slightly decreased (Fig. 5e–g), suggesting that the rest of the electrophysiological features still retain predictive information. However, when SVM was run on each group of variables individually, only features of spikes (MFR and Ch. spikes) got an accuracy over 0.6 for the prediction of Ch. bursts (Fig. 5h). Synchrony and connectivity features kept similar predictive information for STTC (Fig. 5i), whereas prediction of MFR was not accurately performed (accuracy < 0.4) for any group of features (Fig. 5j). Altogether, the cross-validation analysis confirmed that electrophysiological features at DIV 6–8 were highly correlated with the levels of burst and synchronization at the third week in vitro.

## Discussion

Cortical neurons in vitro exhibit along development a wide variety of firing patterns whose descriptive classification and characterization are challenging^10,54^. In this study, we show that the development of neural network activity in cortical neuron cultures recorded by MEAs can be characterized by combining features of spikes, bursts, synchrony, and connectivity. The use of machine learning techniques allowed us to identify clusters of neuronal networks with similar developing spontaneous activity within the range of synchronized and bursting activity in cultured neurons during early development. Indeed, the levels of Ch. bursts, STTC, and MFR at DIV 13–18 could be successfully predicted based on the activity at DIV 6–8 using three different machine learning models, confirming the correlation between the activity in early developmental stages and features of activity in mature neuronal networks.

Our findings are consistent with the average increase in synchronized spiking and bursting activity during the first 3 weeks in vitro reported in previous studies^9–12^. Increases in peak frequency in bursts, probability of burst events (Burst surprise), and percentage of spikes in bursts from DIV 6–8 to DIV 13–18 are consistent with developmental changes toward a network activity dominated by burst activity. Synchronization (STTC) and highly synchronized hubs of neurons (STTC DBSCAN) progressively appeared during the second week in vitro and this type of changes have been previously shown to be strongly correlated with the strengthening of synapses^9,55^, both glutamatergic and GABAergic^56^. The maturation and consequent rate of spontaneous activity in vitro depends also upon the neuronal density of cultures^10,12,14^. The cortical cultures used in this study can be classified as dense cultures (1750–3500 cells/mm^2^) in which bursting activity can be usually observed after only one week in vitro^10^. Therefore, caution should be taken to extrapolate our results to sparse cortical cultures with different network dynamics during early development.

Our results indicate that the analysis of features of spikes, bursts, synchrony, and connectivity may help to characterize the developmental stage of neuronal network activity during the first three weeks in culture. By applying the PCA dimensionality reduction technique to these electrophysiological features, we show that a broad separation of neuronal networks at the first and the third week in vitro could be better accomplished by properties of network activity (e.g., number of active channels or connectivity properties) rather than single features of spikes or bursts. Indeed, the PCA analysis suggested that there may be some redundancy in the information encoded by the 18 electrophysiological features since the two first PC dimensions accounted for approx. 75% of the variance and some network features such as Network spikes and bursts seemed to be strongly correlated.

Our findings also indicated that the variability of activity levels observed in neuronal networks within the same developmental stage, also reported in previous studies^10,12,15^, can be efficiently examined when using clustering analysis across developmental stages. The clusters of neuronal networks defined by the k-means analysis of the SOM based on Ch. bursts (i.e., spatial distribution of burst activity) highly correlated with changes in the four groups of electrophysiological features, whereas STTC clustering was less efficient in characterizing neuronal networks with low-intermediate levels of synchronization and divergent development. This might indicate that neuronal networks with highly isolated burst activity at DIV 6–8 may not always develop strong synchronized burst activity. Thus, Ch. bursts may be used as a predictive marker of network development, considering also that bursts are commonly the consequence of cooperative network activity^57^.

Results obtained in the analysis of functional connectivity suggests that the neuronal networks included in the clusters of high Ch. burst and STTC levels (cluster 1 in both clustering methods) already had properties of small-world topology (high levels of segregation and integration) at 7 DIV, whereas other neuronal networks kept their initial random-like topology at the third week in vitro. Previous studies using 60 electrode MEAs have shown that topology may evolve either from random structure^22^ or from hubs densely interconnected^23^. However, these divergent results may be related to differences in culture densities^21^. It also remains unclear whether synchronized bursts may be triggered by highly active neurons^58^, or bursts may be instead the result of zones with intermediate activity near the network’s boundary as suggested by studies using large-scale MEAs^59^. According to our results, levels of segregation and integration were in general tightly correlated across development. However, as the 60 electrode MEAs cover a partial window of the entire network, the presence of other topologies cannot be totally excluded. Application of clustering techniques to the connectivity analysis of cultured neuronal networks with different cellular densities on high-density electrode arrays may help to elucidate how the emergence of network topology is affected during early development.

Spontaneous activity at early postnatal stages seems to play an essential role in the correct maturation of mammalian cortical networks^5,60^ and machine learning techniques allowed us to further explore the correlation between the activity of immature and mature cortical neuronal networks in vitro. Overall, all three machine learning models (MARS, SVM, and Random Forest) used in this study, efficiently captured the nonlinear changes of network activity that occurred during the second week in vitro and were able to predict with high accuracy the values of Ch. bursts, STTC, and MFR. These results suggest that machine learning techniques might be successfully used as internal control for long-term experiments with developing neuronal networks. Analysis of the importance of the different electrophysiological features in the machine learning models showed that the number of channels with bursts can be predicted with good accuracy even with only two features of spikes (Ch. spikes and MFR) whereas prediction of MFR itself had much lower accuracy, suggesting that the fluctuations in firing rate during development might require a continuous dataset for better prediction. Similarly, the prediction of STTC was less accurate when a lower number of features was used in the SVM model, suggesting that synchronization may require additional features of bursts and connectivity for better characterization. A complementary explanation could be that low and intermediate levels of synchronous activity at DIV 7 may not reflect how synchronization will emerge later in development, as a consequence of recurrent bursting activity^61–63^. In summary, we may argue that, in dense cortical cultures, random spike activity largely distributed across the neuronal network precedes the development of burst activity which may need to reach a certain threshold to become synchronized across the network. Altogether, our clustering and machine learning results suggest that the initial network activity of in vitro neuronal networks may predetermine the consequent developmental trend of network activity.

Due to the somewhat limited dataset and the dependence of network activity on experimental conditions, the interpretation of the results requires caution. Although internal validation of the machine learning models reduces biases and overfitting, further external validation with datasets of similar experimental conditions would reinforce the machine learning results^26^. Moreover, it will be interesting also to explore the influence of parameters such as neuronal survival during the second week in vitro, the balance between excitation and inhibition^64^, or neurite outgrowth^65^ on different developmental patterns of network activity. Nevertheless, the methodological approach presented here for the characterization and early prediction of network activity in cultured neurons may be a useful tool to elucidate the biological causes of variability in network dynamics as well as to optimize the use of cortical cultures in models of health and disease.

## Supplementary Information

Supplementary Information.

## Data Availability

Any supplementary information, code, and source data are available from the corresponding authors (A.N., D.C.-G., and J.V.B.) upon reasonable request.
