# Supplementary material for: Early prediction of developing spontaneous activity in cultured neuronal networks
Source: Sci Rep. 2021 Oct 14;11:20407. doi: 10.1038/s41598-021-99538-9 (PMC8516856; doi:10.1038/s41598-021-99538-9)
Supplement: Supplementary file 1 — Supplementary Information. [file 41598_2021_99538_MOESM1_ESM.docx]

# Supplementary Figures

**Fig. S1. a.** Change in the level of synchronization measured by STTC in relation to the time bin size (3, 10, 100, 200, 500, and 1000 ms). Values are mean ± SEM. **b.** Correlation between the values of STTC (time bin = 100 ms) and the percentage of spikes in bursts. **c.** Efficiency levels were calculated for 3 different time bins (left panel) and threshold levels (right panel). Values are mean ± SEM (left panel) and Tukey box plots (right panel). The figure was created using Graphpad 8.0 (https://[www.graphpad.com](http://www.graphpad.com)).

**
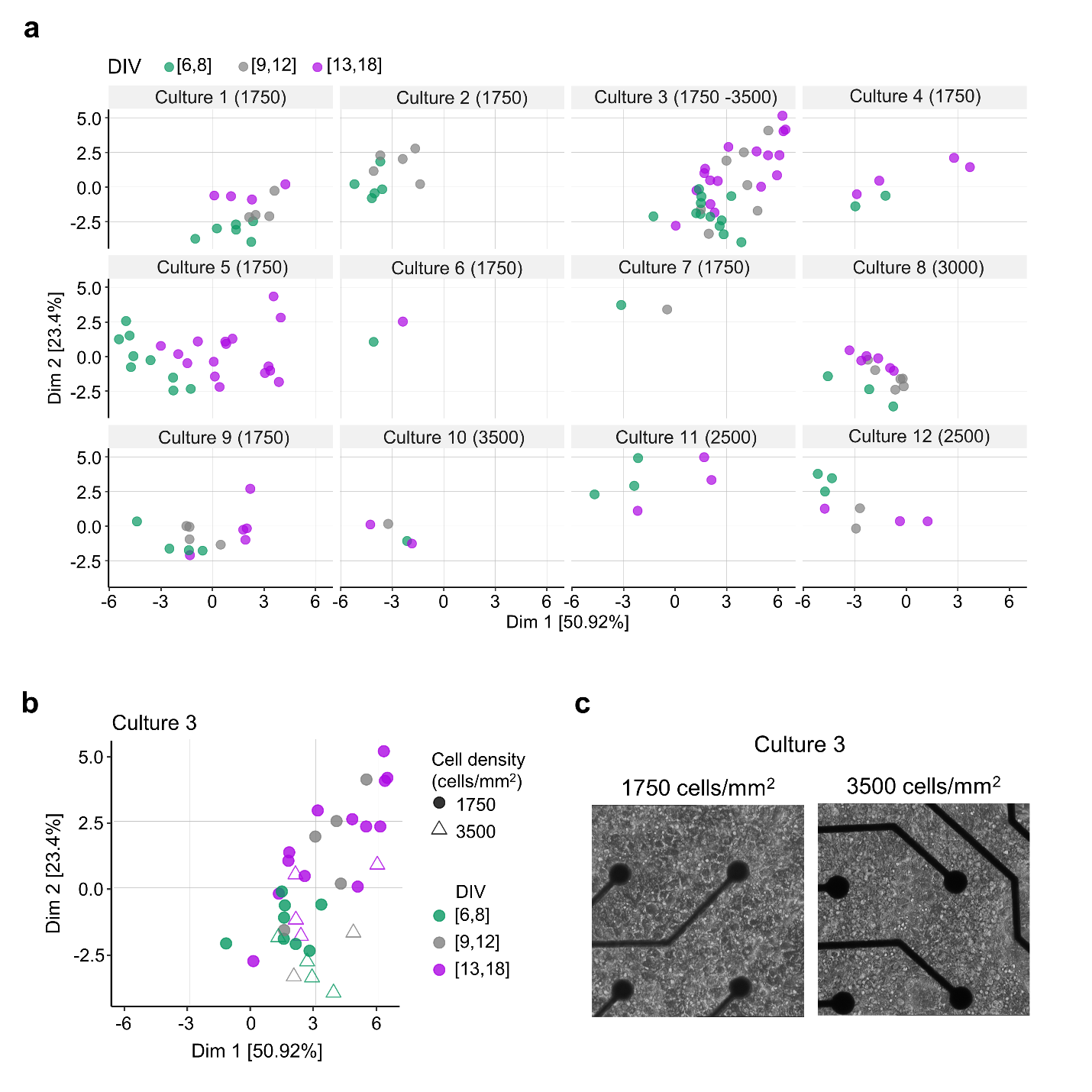
**

**Fig. S2.** Dimensionality reduction of electrophysiological features to differentiate the developmental stage of cortical neurons on MEAs within and between cultures. **a.** PCA projection of 18 electrophysiological features measured in MEA recordings from 12 independent cortical cultures. Neuronal density of the cultures (cells/mm^2^) is indicated in parentheses at the top of each panel. **b.** PCA projection of electrophysiological features of MEA recordings from culture 3 to identify cortical neurons by developmental stage (DIV: 6-8, 9-12, 13-18) and cell density (1750 or 3500 cells/mm^2^). The color of the symbols represents the DIV intervals and cell densities are shown as circles (1750 cells/mm^2^) and triangles (3500 cells/mm^2^). **c.** Phase contrast images from culture 3 showing cortical neurons on MEA dishes at DIV 7 with a density of 1750 cells/mm^2^ (left) and 3500 cells/mm^2^ (right). Distance between electrodes is 200 µm. The figure (**a, b**) was created using R 4.03 (<https://www.r-project.org/>).

**
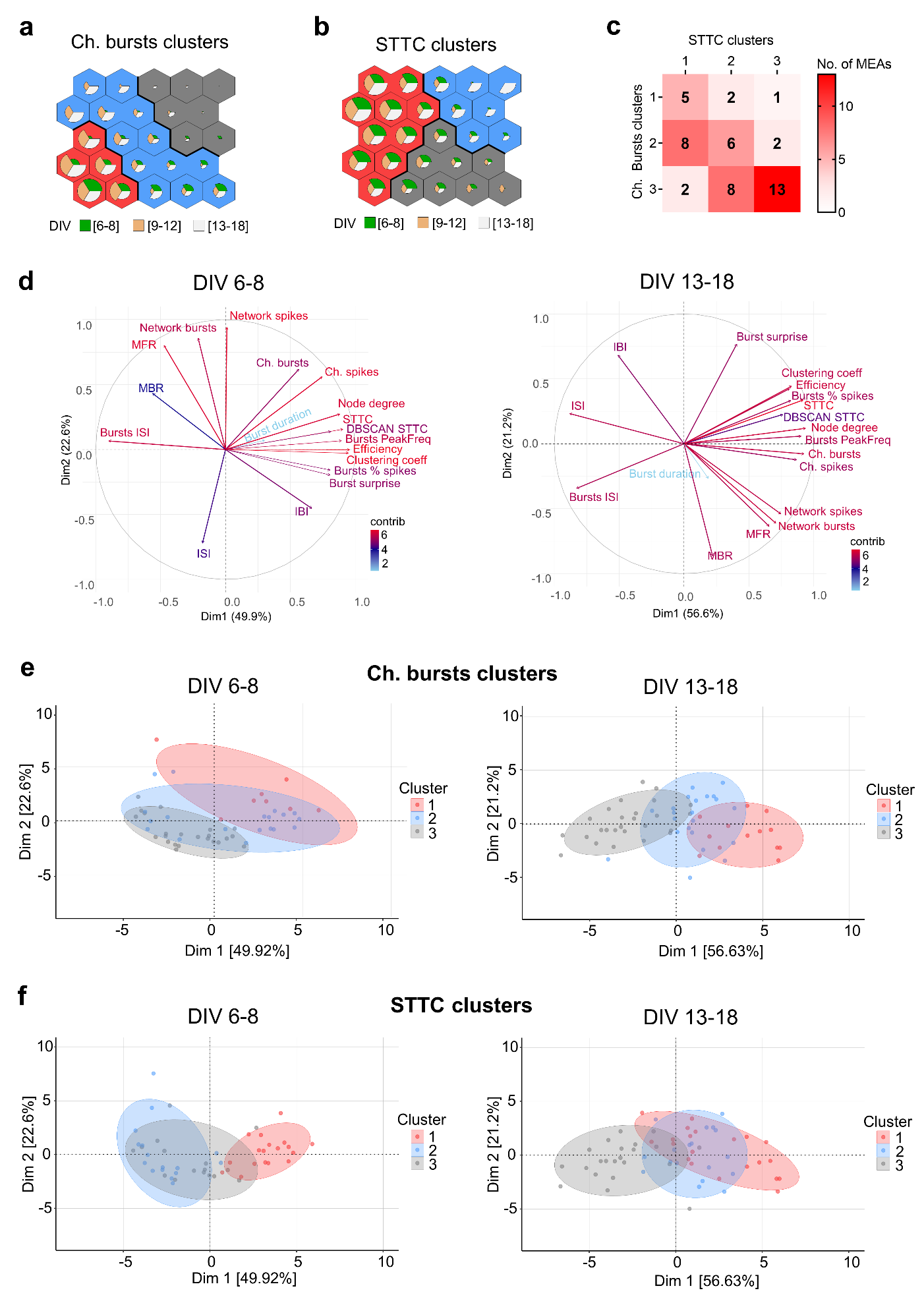
**

**Fig. S3. a-b.** Matrices of the SOM for the variables Ch. bursts (**a**) and STTC (**b**). The SOM matrix with a 5 x 5 hexagonal topology shows the distribution of nodes associated with each of the three k-means clusters. Inside each hexagonal cell (node), pie charts show the values of Ch. bursts (**a**) and STTC (**b**) in each DIV interval: 6-8, 9-12, and 13-18. **c.** Correlation matrix represents the number of neuronal networks (from a total of 47) included in each cluster of Ch. bursts (rows) and STTC (columns). The color in the matrix cells corresponds to the relative value in each cell adjusting the maximum value to 13. **d.** Variable factor map for the PCA of DIV 6-8 (left) and DIV 13-18 (right). Each electrophysiological feature is represented by a vector in the map and the contribution (contrib) of each electrophysiological feature is calculated from their eigenvalues. The vector length and angle with respect to the PC axes represent the contribution and importance of the features in each PC dimension. Angles between vectors also indicate the correlation between features: small angles indicate close relation, 180º means negative correlation while 90º are uncorrelated features. The first 2 PC accounting for 72.5% at DIV 6-8 and 77.5% at DIV 13-18. **e-f**. PCA plots represent recordings at DIV 6-8 (left panels, n = 50) and DIV 13-18 (right panels, n = 61) included in clusters of Ch. bursts (**e**) or STTC (**f**). Colors of dots and PCA confidence ellipsis refer to each cluster of Ch. bursts (**e**) or STTC (**f**). The figure was created using Microsoft PowerPoint 365 (<https://www.microsoft.com/powerpoint>), R 4.0 (<https://www.r-project.org/>), and Graphpad 8.0 (https://[www.graphpad.com](http://www.graphpad.com)).


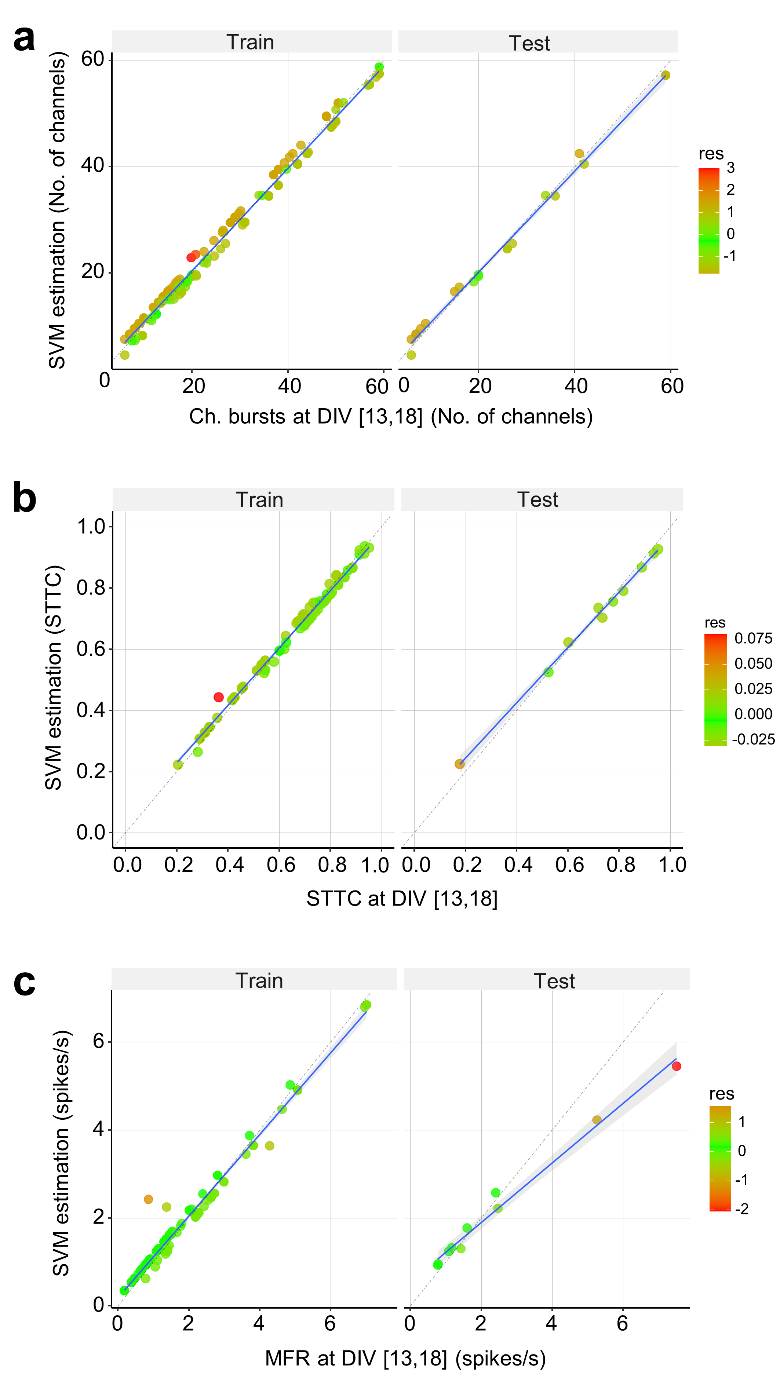


**Fig. S4.** Scatter plots of the SVM prediction. **a-c**. The graphs show the estimation values of the SVM model (y-axis) in relation to the measured values at DIV 13-18 (x-axis) for the electrophysiological features of Ch. bursts (**a**), STTC (**b**), MFR (**c**). Train (left panels) and test (right panels) results of the SVM model are shown. Gray area represents the 95% confidence interval, and the dotted line represents perfect estimation. The color of the dots show the value of the residuals. Values of accuracy and RMSE for the SVM model are listed in Table S4. The figure was created using R 4.0 (https://www.r-project.org/).

| **Table S1. Description of electrophysiological features used to characterize network development of cortical neurons on MEAs.** | | |
| --- | --- | --- |
| **Features** | **Abbreviation** | **Description** |
| Channels with spikes | Ch. spikes | Number of channels recording spike activity (maximum of 59 channels). |
| Network spikes | Network spikes | Sum of spikes/s in all channels with spikes. |
| Mean firing rate | MFR | Mean number of spikes per second. |
| Interspike interval | ISI | Mean interval (s) between spikes. |
| Channels with bursts | Ch. bursts | Number of channels recording burst activity (maximum of 59 channels). |
| Network bursts/min | Network bursts | Sum of bursts/min in all channels with bursts. |
| Mean bursting rate | MBR | Mean value of bursts/min in each MEA. |
| Burst duration | Burst duration | Mean duration of the bursts in seconds. |
| Percentage of spikes in bursts | Burst % spikes | Percentage of spikes in bursts in comparison with the total number of spikes. |
| Interspike interval in bursts | Burst ISI | Mean interval (s) between spikes within bursts. |
| Peak frequency in bursts | Burst PeakFreq | Mean of the inverse of the minimum interspike interval in a burst (Hz). |
| Interburst interval | IBI | Mean interval (s) between the last spike in a burst and the first spike in the next burst on the same channel. |
| Burst surprise | Burst surprise | Mean burst surprise based on S = -log10 (P), where P is the probability that P has at least N spikes during the burst duration. |
| Spike Time Tiling Coefficient | STTC | STTC quantifies the pairwise correlation between spike trains from 2 channels. Values from 0 to 1. |
| DBSCAN clustering in STTC | STTC DBSCAN | Density-based spatial clustering of applications with noise (DBSCAN) algorithm applied to STTC cluster channels with similar levels of synchronization. |
| Average node degree | Node degree | The average node degree measures the mean number of links connected to an electrode (node). It estimates the density of connections of the network. Values from 1 to 58. |
| Clustering coefficient | Clustering coeff | Fraction of triangles around a node and is equivalent to the fraction of node’s neighbors that are neighbors of each other. It estimates the segregation of the network. Values from 0 to 1. |
| Global efficiency | Efficiency | The average inverse shortest path length in the network and estimates the level of integration of the network. Values from 0 to 1. |

Black horizontal lines separate groups of features (from top to bottom): spikes, bursts, synchrony, and connectivity.

| **Table S2. P-values for the differences between electrophysiological features in DIV intervals.** | | | | |
| --- | --- | --- | --- | --- |
| **Electrophysiological features** | All DIV intervals | DIV 6-8 vs 9-12 | DIV 6-8 vs 13-18 | DIV 9-12 vs 13-18 |
| Ch. spikes | **<0.0001** | 0.1266 | **<0.0001** | 0.2703 |
| Network spikes | **<0.0001** | **0.0028** | **<0.0001** | 0.2353 |
| MFR | **<0.0001** | **0.0018** | **<0.0001** | 0.5709 |
| ISI | **0.0041** | 0.0528 | **0.0050** | >0.9999 |
| Ch. Bursts | **<0.0001** | **0.0007** | **<0.0001** | 0.5555 |
| Network bursts | **<0.0001** | **0.0017** | **<0.0001** | 0.0851 |
| MBR | **<0.0001** | 0.1671 | **<0.0001** | 0.1710 |
| Burst duration | 0.0899 | >0.9999 | 0.2955 | 0.1488 |
| Burst % spikes | **0.0131** | 0.3207 | **0.0101** | >0.9999 |
| Burst ISI | **<0.0001** | 0.6038 | **<0.0001** | **0.0343** |
| Burst Peak Freq. | **0.0001** | **0.0494** | **<0.0001** | 0.6991 |
| IBI | **<0.0001** | 0.4768 | **<0.0001** | **0.0136** |
| Burst surprise | 0.5805 | >0.9999 | 0.9626 | >0.9999 |
| STTC | **0.0025** | 0.4664 | **0.0016** | 0.4127 |
| STTC DBSCAN | **0.0098** | 0.3624 | **0.0072** | 0.9686 |
| Node degree | **0.0004** | 0.1769 | **0.0002** | 0.4407 |
| Clustering Coeff | **0.0079** | 0.4293 | **0.0056** | 0.7559 |
| Efficiency | **0.0066** | 0.4692 | **0.0046** | 0.6436 |
| P-values by Kruskal Wallis and Dunn’s post hoc multiple comparison test. P-values < 0.05 are highlighted in bold. | | | | |

| **Table S3. Values of electrophysiological features for clusters of Ch. bursts and STTC.** | | | | | | | | | |
| --- | --- | --- | --- | --- | --- | --- | --- | --- | --- |
|  | **Ch. bursts** | | | | | |  |  |  |
| **Electrophysiological**  **features** | **Cluster 1** (n = 8) | | **Cluster 2** (n = 16) | | **Cluster 3** (n = 23) | | **P-value** | |  |
|  | DIV 6-8 | DIV 13-18 | DIV 6-8 | DIV 13-18 | DIV 6-8 | DIV 13-18 | DIV 6-8 | DIV 13-18 |  |
| Ch. spikes (n) | 48.5 ± 3.0 | 56.4 ± 1.0 | 28.8 ± 2.7 | 44.9 ± 1.1 | 9.4 ± 1.1 | 20.2 ± 1.3 | **<0.0001** | **<0.0001** |  |
| Network spikes/s | 48.9 ± 12.3 | 223.0 ± 33.4 | 20.5 ± 5.2 | 92.2 ± 18.9 | 6.7 ± 1.6 | 24.8 ± 4.2 | **<0.0001** | **<0.0001** |  |
| MFR | 1.1 ± 0.3 | 3.9 ± 0.6 | 0.8 ± 0.2 | 2.0 ± 0.4 | 0.7 ± 0.2 | 1.2 ± 0.1 | **0.0004** | **<0.0001** |  |
| ISI (s) | 2.4 ± 0.3 | 1.2 ± 0.2 | 4.2 ± 0.4 | 2.9 ± 0.2 | 5.5 ± 0.4 | 4.7 ± 0.3 | **<0.0001** | **<0.0001** |  |
| Ch. bursts (n) | 26.9 ± 5.1 | 53.4 ± 1.7 | 14.4 ± 2.5 | 37.5 ± 1.4 | 3.7 ± 0.8 | 13.6 ± 1.2 | **0.0423** | **<0.0001** |  |
| Network bursts/min | 175.0 ± 76.4 | 554.5 ± 56.2 | 67.0 ± 22.4 | 286.5 ± 36.6 | 29.8 ± 8.8 | 91.4 ± 12.4 | **0.0007** | **<0.0001** |  |
| MBR | 7.1 ± 2.8 | 10.6 ± 1.1 | 5.9 ± 2.0 | 7.8 ± 1.0 | 8.7 ± 3.0 | 6.7 ± 0.7 | 0.5659 | **0.0480** |  |
| Burst duration (ms) | 420.9 ± 54.7 | 380.0 ± 50.0 | 471.8 ± 87.1 | 290.4 ± 31.8 | 331.6 ± 23.7 | 337.8 ± 31.5 | 0.3211 | 0.3036 |  |
| Burst % spikes | 70.9 ± 5.9 | 89.1 ± 2.4 | 69.1 ± 6.6 | 80.2 ± 2.6 | 60.4 ± 3.5 | 70.2 ± 2.6 | 0.1484 | **<0.0001** |  |
| Burst ISI (ms) | 33.5 ± 6.3 | 23.4 ± 2.0 | 46.2 ± 5.1 | 29.1 ± 2.0 | 59.4 ± 3.4 | 41.1 ± 2.3 | **0.0072** | **<0.0001** |  |
| Burst PeakFreq | 197.2 ± 22.6 | 309.2 ± 21.1 | 169.7 ± 18.1 | 225.6 ± 10.8 | 94.9 ± 13.4 | 162.8 ± 9.8 | **0.0015** | **<0.0001** |  |
| IBI (s) | 26.9 ± 5.3 | 8.1 ± 0.9 | 27.7 ± 3.5 | 13.8 ± 1.1 | 24.6 ± 2.9 | 17.1 ± 1.4 | 0.9237 | **0.0001** |  |
| Burst surprise | 43.6 ± 10.3 | 27.4 ± 4.2 | 42.5 ± 7.8 | 30.7 ± 4.6 | 25.1 ± 4.4 | 20.8 ± 2.8 | 0.1526 | 0.2756 |  |
| STTC | 0.6 ± 0.1 | 0.8 ± 0.0 | 0.5 ± 0.1 | 0.7 ± 0.0 | 0.3 ± 0.0 | 0.5 ± 0.0 | **0.0077** | **<0.0001** |  |
| STTC DBSCAN | 0.6 ± 0.1 | 0.9 ± 0.0 | 0.6 ± 0.1 | 0.8 ± 0.0 | 0.2 ± 0.1 | 0.5 ± 0.1 | **0.0050** | **<0.0001** |  |
| Node degree | 38.5 ± 7.1 | 50.3 ± 2.8 | 18.5 ± 3.9 | 32.8 ± 2.7 | 2.5 ± 0.7 | 8.1 ± 1.3 | **0.0002** | **<0.0001** |  |
| Clustering coeff | 0.8 ± 0.1 | 0.9 ± 0.0 | 0.6 ± 0.1 | 0.9 ± 0.0 | 0.3 ± 0.1 | 0.6 ± 0.1 | **0.0019** | **<0.0001** |  |
| Efficiency | 0.8 ± 0.1 | 0.9 ± 0.0 | 0.6 ± 0.1 | 0.8 ± 0.0 | 0.3 ± 0.1 | 0.5 ± 0.1 | **0.0240** | **<0.0001** |  |
|  | **STTC** | | | | | |  |  |  |
| **Electrophysiological**  **features** | **Cluster 1** (n = 15) | | **Cluster 2** (n = 16) | | **Cluster 3** (n = 16) | | **P-value** | |  |
|  | DIV 6-8 | DIV 13-18 | DIV 6-8 | DIV 13-18 | DIV 6-8 | DIV 13-18 | DIV 6-8 | DIV 13-18 |  |
| Ch. spikes (n) | 37.7 ± 3.3 | 47.9 ± 2.6 | 13.3 ± 2.8 | 41.8 ± 2.6 | 16.3 ± 3.3 | 22.2 ± 2.5 | **<0.0001** | **<0.0001** |  |
| Network spikes/s | 20.3 ± 3.8 | 142.4 ± 29.7 | 20.2 ± 8.4 | 96.1 ± 18.8 | 15.2 ± 5.9 | 38.8 ± 15.0 | 0.0618 | **0.0003** |  |
| MFR | 0.5 ± 0.1 | 2.7 ± 0.5 | 1.3 ± 0.3 | 2.2 ± 0.3 | 0.8 ± 0.2 | 1.4 ± 0.3 | 0.2514 | 0.0693 |  |
| ISI (s) | 3.8 ± 0.4 | 2.5 ± 0.4 | 4.8 ± 0.6 | 2.5 ± 0.2 | 5.1 ± 0.5 | 4.6 ± 0.3 | 0.1079 | **0.0001** |  |
| Ch. bursts (n) | 19.3 ± 3.1 | 42.6 ± 3.1 | 5.9 ± 2.1 | 36.1 ± 2.7 | 8.2 ± 2.7 | 15.0 ± 2.3 | **0.0240** | **<0.0001** |  |
| Network bursts/min | 49.5 ± 12.6 | 352.5 ± 55.3 | 117.2 ± 54.5 | 324.6 ± 50.4 | 51.1 ± 19.5 | 120.2 ± 28.8 | 0.6793 | 0.0001 |  |
| MBR | 2.7 ± 0.8 | 7.7 ± 1.0 | 13.0 ± 3.1 | 9.0 ± 1.2 | 8.0 ± 3.6 | 7.2 ± 0.8 | 0.1270 | 0.7455 |  |
| Burst duration (ms) | 467.9 ± 85.1 | 317.2 ± 39.3 | 318.8 ± 32.4 | 302.8 ± 25.6 | 391.0 ± 46.5 | 363.7 ± 39.6 | 0.1931 | 0.5203 |  |
| Burst % spikes | 85.0 ± 2.4 | 86.8 ± 2.2 | 45.1 ± 4.4 | 80.9 ± 2.4 | 60.2 ± 4.5 | 66.8 ± 2.8 | **<0.0001** | **0.0001** |  |
| Burst ISI (ms) | 30.0 ± 2.3 | 23.5 ± 1.1 | 66.5 ± 4.6 | 30.0 ± 1.8 | 58.9 ± 3.7 | 44.5 ± 2.6 | **<0.0001** | **<0.0001** |  |
| Burst PeakFreq | 218.7 ± 11.7 | 276.6 ± 17.7 | 83.7 ± 12.6 | 226.3 ± 12.8 | 100.0 ± 14.9 | 151.8 ± 8.7 | **<0.0001** | **<0.0001** |  |
| IBI (s) | 34.2 ± 3.0 | 12.6 ± 1.7 | 15.5 ± 2.4 | 12.7 ± 1.5 | 25.8 ± 3.6 | 16.4 ± 1.3 | 0.0016 | **0.0376** |  |
| Burst surprise | 57.4 ± 6.0 | 31.3 ± 3.8 | 11.5 ± 4.2 | 30.5 ± 5.3 | 28.6 ± 5.5 | 16.7 ± 2.3 | **<0.0001** | **0.0258** |  |
| STTC | 0.7 ± 0.0 | 0.8 ± 0.0 | 0.2 ± 0.0 | 0.7 ± 0.0 | 0.4 ± 0.0 | 0.4 ± 0.0 | **<0.0001** | **<0.0001** |  |
| STTC DBSCAN | 0.8 ± 0.0 | 0.9 ± 0.0 | 0.1 ± 0.1 | 0.8 ± 0.0 | 0.3 ± 0.1 | 0.4 ± 0.1 | **<0.0001** | **<0.0001** |  |
| Node degree | 33.9 ± 3.7 | 41.5 ± 3.2 | 1.5 ± 0.1 | 31.2 ± 3.0 | 5.0 ± 2.3 | 6.9 ± 2.1 | **<0.0001** | **<0.0001** |  |
| Clustering coeff | 0.9 ± 0.0 | 0.9 ± 0.0 | 0.1 ± 0.1 | 0.9 ± 0.0 | 0.4 ± 0.1 | 0.5 ± 0.1 | **<0.0001** | **<0.0001** |  |
| Efficiency | 0.9 ± 0.0 | 0.9 ± 0.0 | 0.1 ± 0.1 | 0.8 ± 0.1 | 0.3 ± 0.1 | 0.4 ± 0.1 | **<0.0001** | **<0.0001** |  |
| The number of MEAs included in each cluster (n) is shown on the top row. Values are mean ± SEM. Kruskal-Wallis test was used to compare differences in electrophysiological features between clusters in each DIV interval. P-values < 0.05 in bold font. | | | | | | | | | |

| **Table S4. Performance results of machine learning models for the variables Ch. bursts, STTC and MFR.** | | | | | | | | | | | |
| --- | --- | --- | --- | --- | --- | --- | --- | --- | --- | --- | --- |
|  | **Ch. bursts** | | |  | **STTC** | | |  | **MFR** | | |
| **Performance** | MARS | SVM | RF |  | MARS | SVM | RF |  | MARS | SVM | RF |
| Training (R^2^) | 0.995 | 0.991 | 0.996 |  | 0.993 | 0.989 | 0.942 |  | 0.997 | 0.975 | 0.978 |
| Test (R^2^) | 0.994 | 0.993 | 0.981 |  | 0.970 | 0.986 | 0.916 |  | 0.904 | 0.875 | 0.817 |
| Test (RMSE) | 1.233 | 1.379 | 2.253 |  | 0.038 | 0.025 | 0.064 |  | 0.652 | 0.746 | 0.900 |
| Root mean squared error (RMSE). RF: Random Forest. | | | | | | | | | | | |

| **Table S5. Relative importance of electrophysiological features in each machine learning model for the prediction of Ch. bursts, STTC and MFR at DIV 13-18.** | | | | | | | | | | | |
| --- | --- | --- | --- | --- | --- | --- | --- | --- | --- | --- | --- |
|  | **Ch. bursts** | | |  | **STTC** | | |  | **MFR** | | |
| **Features** | MARS | SVM | RF |  | MARS | SVM | RF |  | MARS | SVM | RF |
| Ch. spikes * | **100.0** | **100.0** | **100.0** |  | **93.1** | 62.9 | 29.9 |  | 43.5 | 79.9 | 66.3 |
| Network spikes * | 31.2 | **89.8** | 19.6 |  | 37.4 | 4.2 | 3.4 |  | 39.3 | **100.0** | 67.9 |
| MFR | 44.9 | 26.4 | 0.0 |  | n/a | 0.0 | 1.0 |  | n/a | 23.5 | 5.0 |
| ISI | n/a | 56.3 | 19.2 |  | 83.5 | 16.0 | 23.7 |  | **100.0** | **90.7** | **100.0** |
| Ch. bursts * | 40.7 | 64.5 | 13.7 |  | n/a | 18.4 | 0.0 |  | 42.0 | 88.1 | **38.8** |
| Network bursts * | n/a | 31.1 | 2.6 |  | **93.1** | 15.6 | 1.4 |  | n/a | 75.2 | 31.1 |
| MBR | n/a | 24.8 | 2.0 |  | 93.1 | 6.2 | 10.0 |  | 49.6 | 14.6 | 5.2 |
| Burst duration | n/a | 21.7 | 2.4 |  | 55.6 | 1.5 | 2.9 |  | **100.0** | 36.5 | **69.6** |
| Burst % spikes | **64.9** | 22.8 | 20.6 |  | n/a | 23.0 | 19.7 |  | **62.6** | 0.0 | 0.0 |
| Burst ISI | 38.9 | 57.1 | 7.4 |  | n/a | 74.4 | 28.2 |  | 49.6 | 33.4 | 0.0 |
| Burst PeakFreq | n/a | 35.7 | 5.9 |  | n/a | 80.3 | **61.9** |  | 33.6 | 6.8 | 7.5 |
| IBI | **53.0** | 16.9 | 7.8 |  | 15.8 | 48.4 | 16.5 |  | 46.6 | 3.8 | 14.2 |
| Burst surprise | 16.9 | 0.0 | 7.6 |  | 15.8 | 11.1 | 9.2 |  | 36.1 | 2.3 | 12.8 |
| STTC * | 36.2 | 36.9 | 10.0 |  | 55.6 | 79.2 | 28.0 |  | n/a | 9.2 | 4.5 |
| STTC DBSCAN * | n/a | 20.7 | 0.6 |  | 20.2 | 68.0 | 54.8 |  | 49.6 |  | 12.4 |
| Node degree | n/a | **89.9** | **51.9** |  | **100.0** | **100.0** | **100.0** |  | 10.7 | **80.7** | **91.0** |
| Clustering coeff * | 12.9 | 64.3 | 9.6 |  | **93.1** | **86.9** | 44.9 |  | 49.6 | 29.6 | 12.3 |
| Efficiency * | n/a | 51.1 | 5.4 |  | 56.9 | **81.0** | **60.2** |  | 49.6 | 8.8 | 6.0 |
| Values in bold are the three most important features for prediction in each machine learning model. * Electrophysiological features used for leave-one-in and leave-one-out cross-validation in **Fig. 5e-j**. Black horizontal lines separate groups of features (from top to bottom): spikes, bursts, synchrony, and connectivity. RF: Random Forest. | | | | | | | | | | | |
